# Monte Carlo-based dosimetric comparison of indigenous and commercial ^106^Ru/^106^Rh eye plaques using a mathematical eye phantom

**DOI:** 10.1007/s12194-025-00986-z

**Published:** 2025-11-18

**Authors:** Vandana Shrivastava, Arghya Chattaraj, T. Palani Selvam, M. S. Pathan, Rajesh Kumar, B. K. Sapra

**Affiliations:** 1 Radiological Physics & Advisory Division, Medical Group, Bhabha Atomic Research Centre, Trombay, Mumbai, 400 085 India; 2 Homi Bhabha National Institute, Anushaktinagar, Mumbai, 400 094 India

**Keywords:** ^106^Ru/^106^Rh eye plaque, Beta particle, Monte carlo, Eye dosimetry

## Abstract

This study evaluates and compares the dosimetric performance of the Bhabha Atomic Research Centre (BARC)^106^Ru/^106^Rh eye plaques and a commercially available similar BEBIG CCA eye plaque using Monte Carlo simulations via the FLUKA code. Simulations were performed in both spherical water and mathematical eye phantoms to determine dosimetric parameters such as reference absorbed dose rates, percentage depth doses, off-axis dose profiles, and 2D dose mappings. The on-axis reference absorbed dose rates at 1 mm depth in the mathematical eye phantom was calculated as 10.712 ± 0.003 mGy/min-MBq for the BEBIG CCA plaque and 10.194 ± 0.003 mGy/min-MBq for the BARC plaque, showing close agreement between the two applicators. Off-axis and depth dose distributions confirmed that the BARC plaque delivers comparable dose profiles to the BEBIG plaque, with all critical eye structure doses, including the lens, retina, and cornea, well within established tolerance limits. The similarity in dose distributions between water and mathematical phantoms suggests that water can serve as a practical approximation for dosimetry evaluations. This study demonstrates that the BARC plaque provides dosimetric characteristics comparable to the BEBIG plaque, support its potential use in clinical ophthalmic brachytherapy.

## Introduction

In the past, the intraocular cancers, particularly conditions such as malignant melanoma and uvea tumours used to be effectively managed by enucleation (complete removal of eye ball). However, utilization of brachytherapy for the treatment of intraocular cancer has emerged as a favoured approach over the more invasive enucleation procedure [1–3]. Choroidal melanoma predominantly afflicts adults, whereas retinoblastoma is predominantly observed in children. Collaborative Ocular Melanoma Study (COMS) proved that plaque brachytherapy provides additional advantages of eye and vision preservation along with clinical outcome offered by enucleation [4–7].

The classifications of eye tumours are primarily based on the tumour’s thickness and the maximum diameter of its base, following the guidelines outlined in ICRU 72 [3]. Small tumours (T1) are defined by a thickness ranging from 1 to 3 mm and a maximum tumour dimension of the tumour basis up to 10 mm. Medium-sized tumours (T2) exhibit a thickness between 3 and 5 mm, with the greatest tumour dimension of the tumour basis falling within the range of 10–15 mm. In contrast, large-sized tumours (T3) are characterized by a thickness exceeding 5 mm and a maximum tumour dimension of the tumour basis greater than 15 mm [3].

Choice of treatment for these tumours hinges on tumour size, height of the tumour apex, and its precise location[3]. These parameters subsequently guides the selection of an appropriate plaque size and radio-nuclide for brachytherapy treatment[1, 2]. ^103^Pd and ^125^I applicators are considered as the most suitable choices for large-sized tumours [2,4- 10]. Whereas ^06^Ru/^106^Rh beta applicators are commonly preferred [3, 11, 12] for treatment of melanomas of medium sized tumours due to their physical properties of beta radiation, which exhibits a short range, resulting in a substantial dose drop-off over distance and lower radiation exposure to surrounding healthy tissues.

The dosimetry parameters required for ophthalmic treatment are: (a) absolute dose rate to tissue or water at the surface of the applicator and at a certain reference depth along the central axis of the source perpendicular to the surface; (b) on-axis relative depth dose distribution in tissue or water close to the source; and (c) relative dose distribution as a function of off-axis position from the central axis [13]. According to ICRU report 72[3], the recommended method for specifying the radiation intensity of all therapeutic beta-particle sources is the reference absorbed-dose rate, which refers to the absorbed-dose rate to water at a specified reference point. In the case of beta planar and concave sources, the reference point is located at a distance of 1 mm from the centre of the source surface along the central axis.

A variety of commercial beta applicators—CCA, CCB, CCC, CCD, CCX/Y/Z, CXS, CGD, CIA, and CIB/CIB2 are available for ocular therapy [14]. Determination of dose distributions is challenging because of steep gradients over distance and the dimension of the detector [14]. Dosimetry using physical detector is time-intensive, prone to positioning errors and requires accurate calibration. Two approaches are commonly used for dosimetric calculations[14]: (a) Monte Carlo simulations, which model radiation transport with high accuracy; and (b) analytical point-kernel methods, which provides an estimation of the dose distributions but are less precise.

Several Monte Carlo–based studies on dosimetry of^106^Ru/^106^Rh plaques are available in the literatures [4, 12, 14–17]. Hermida-López and Brualla[15] used PENELOPE to study the impact of the^106^Rh gamma spectrum using different plaque models such as CCA, CCC, CCX, CIA and showed its negligible contribution to eye dose. Barbosa et al. [12] employed MCNPX to calculate doses for CCA and CCB plaques in both AAPM and ABS TG-129 recommended mathematical eye phantom and a water phantom, comparing heterogeneous and homogeneous models. Mostafa et al.[16] used GATE to evaluate dose distributions from planar and concave plaques, highlighting the power of the Monte Carlo technique for precise dose calculations with different shapes of eye applicators. Mowlavi and Yazdani [17] applied MCNP4C to obtain isodose curves and dosimetric data for CCA and CCB plaques in a mathematical eye model.

Recently, Rajesh Kumar et al.[4]carried out dosimetry for indigenously developed round type BARC ^106^Ru/^106^Rh eye plaque.Using radiochromic film and diode detector in a custom phantom, they measured absorbed dose rate to water at various on-axis depths and non-uniformity of the activity distribution.Monte Carlo calculations with egs_brachy (part of EGSnrc Monte Carlo code system**)** confirmed measured depth-dose data.The measured depth-dose were also compared against manufacturer certificate values for CCA plaques. However, for treatment planning additional data such as off-axis dose distribution as a function of depth and 2D dose mapping at a plane are also required.

The present study addresses this gap area. The objective of this study is to conduct a comprehensive dosimetry in a mathematical eye phantom for BARC ^106^Ru/^106^Rh eye plaque and CCA eye plaque using FLUKA Monte Carlo code [18, 19]. The study includes calculations of absorbed dose rate at depths of 1 and 2 mm, percentage depth dose, off-axis dose profiles and 2D dose mapping. Furthermore, the study also presents doses to critical eye structures, namely, the eye lens, retina, choroid, and cornea. These dose data calculated for BARC eye plaque are compared with the corresponding data of CCA eye plaque.

## Materials and methods

### ^106^Ru/^106^Rh radionuclide

The ruthenium ophthalmic applicator contains radionuclide^106^Ru which is a pure β emitter with maximum energy of 39.4 keV and mean energy of 10 keV. Its half-life is 368.2 days and it disintegrates to ^106^Pd via ^106^Rh. The daughter ^106^Rh is also a beta source having maximum and mean energies as 3.54 MeV and 1.412 MeV, respectively, providing the effective therapeutic irradiation [20, 21]. It is also emitting 0.34 photons per beta disintegration. The mean energy of gamma radiation is 0.600 MeV, where the greatest yield belongs to 0.512, 0.622, and 1.05 MeV transitions [20, 21]. The half-life of ^106^Rh is 29.9 s. The combined beta spectrum of ^106^Ru and ^106^Rh is utilised in the simulations [21].

### Description of ^106^Ru/^106^Rh ophthalmic applicators

This study incorporates two ^106^Ru/^106^Rh ophthalmic applicators, namely: CCA concave, manufactured by Eckert & Ziegler BEBIG GmbH and BARC eye plaque. CCA BEBIG model is a truncated spherical shell structure with 12.0 mm inner radius of the shell, along the symmetry axis. The outer and active diameters are 15.3 mm and 13 mm, respectively [4, 12, 22]. It is made of 3 layers with total thickness of 1.0 mm. These layers are made of silver having thicknesses of 0.1, 0.2 and 0.7 mm for front, middle and back layers, respectively. BARC eye plaque is almost similar to the CCA concave plaque having made of three layers (front, middle and back) of concentric spheres [4]. These layers are made of silver having thicknesses of 0.1, 0.2 and 0.7 mm for front, middle and back layers, respectively, and the total thickness is 1 mm. The outer diameter, active diameter and radius of curvature are 15.8 mm, 13.3 mm and 12 mm, respectively [4]. Table 1 presents the geometrical parameters of ^106^Ru/^106^Rh for CCA and BARC eye plaque.

**Table 1:** Details on geometric parameters of ^106^Ru/^106^Rh CCA and BARC eye plaque [4, 12]

Type	Manufacturer & Supplier	Diameter D (mm)	Active Diameter D_a_ (mm)	Radius of curvature *R*_c_ (mm)	
CCA	BEBIG	15.3	13	12	
Round	BARC/BRIT^*^	15.8	13.3	12	

^*^Board of Radiation in Isotope Technology

### Anatomy of the human eye

The detailed description of each components of the eye anatomy is discussed elsewhere [23–25]. Here, a brief description is included. The eye is composed of three layers enclosing the eye body, namely the retina (inner), choroids (middle) and the sclera (outer). According to the ICRP 23 [23–25] the total weight of both eyes is 15 g. Both sclera and choroid are 1 mm thick on an average. The critical parts for vision are the retina, macula, lens, optic nerve and disc. The sclera is the tough white fibrous outer layer, the retina in the globe and the uvea is middle vascular layer which consists of iris, choroid, and ciliary body. The iris is the coloured muscle with a small opening that forms the pupil. The choroid is a thin and pigmented layer containing capillaries. The ciliary body contains the ciliary muscles that change the shape of the lens to adjust the focus of the eye. The third and inner layer of the globe (sensory layer) is the retina, composed of specialized light sensitive nerve cells which are connected to the optic nerve. The remainder of the eye is filled with fluid and is called the vitreous body. The orbit of the eye consists of tissues surrounding the globe, and includes the muscles and optic nerve attached to the eye. The structures around the eye including the eyelids and tear glands are adnexal structures [23–25].

### Description of mathematical eye phantom

Human eye and its components such as sclera, choroid, retina, tumour, cornea, eye lens, anterior chamber and vitreous body are modelled mathematically. The sclera, choroid and retina are modelled as three concentric spherical shells and the tumour is an ellipsoid cut by the sclera spherical surface forming a semi-ellipsoid which is situated inside the inner most part of the eye [17]. The cornea is an elliptical shell limited by two concentric ellipses and the outer sclera spherical surface and the lens is formed by the region surrounded by the spherical surface of the sclera and the elliptical surface. The anterior chamber is the geometric region between the surfaces of the inner wall of the cornea and the outer surface of the sclera. The vitreous body is the spherical region limited by the inner surface of the retina. For more details on mathematical eye phantom refer the publication of by Mowlavi and Yazdani [17]. The material densities and elemental compositions of various eye organs are as per the published literature [12]. Table 2 shows the density and elemental composition of each eye region [12].

### FLUKA calculations

**Fig. 1:**
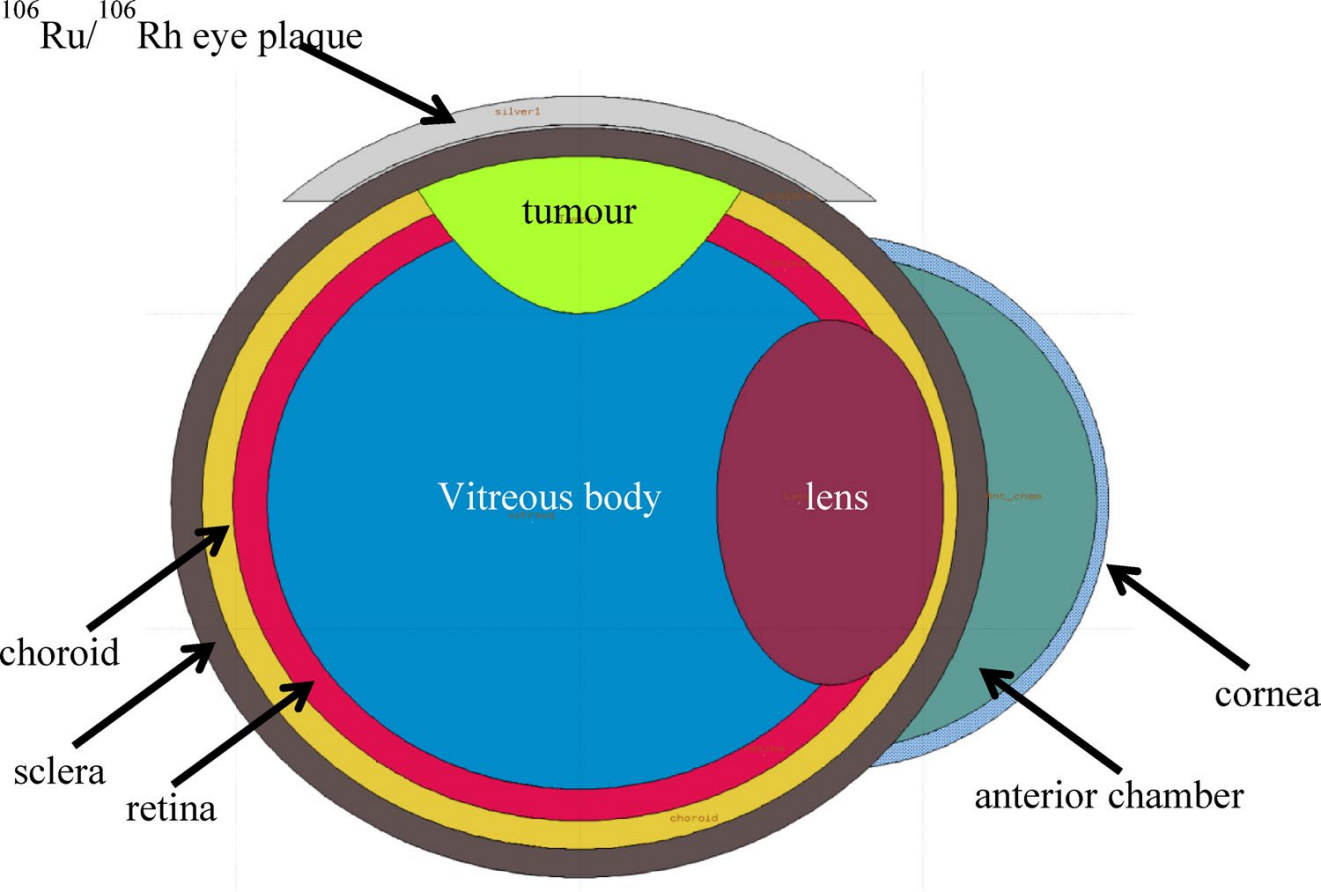
Mathematical eye model with ^106^Ru/^106^Rh eye plaque in FLUKA

Several Monte Carlo codes such as FLUKA, EGSnrc and, MCNPX, and GATE are available for radiation transport simulations; however, modeling of spherical geometry is not supported in the EGSnrc code. Although FLUKA, GATE, and MCNPX can handle such geometries, MCNPX is not an open-source. Between FLUKA and GATE, we selected FLUKA for the present study due to its versatility and ease of use through the FLAIR-based graphical user interface (GUI), which facilitates accurate and efficient modeling of complex geometries. FLUKA is an open-source, general-purpose Monte Carlo code for particle transport in matter, and its physics models have been extensively benchmarked for medical dosimetry applications [26–28]. FLUKA has also been widely applied in beta-particle dosimetry studies [26, 27]. In the study CCA and BARC plaques are simulated using the FLUKA code (version2011.2c) [18, 19], by defining their geometrical structures through the grouping of quadric surfaces such as spheres and planes. Using FLUKA, the mathematical eye phantom with different structures of eye namely sclera, choroid, retina, tumour, cornea, eye lens, anterior chamber and vitreous body are modeled. For both the plaques, the thickness of active layer is considered as 1 μm and it is confined in the middle layer of the plaque. The uniform random sampling of the beta particles emitting isotropically from active layer of the plaque was carried out using FORTRAN-based source.f script. Also note that during simulation, a uniform activity distribution is considered in both eye plaques, whereas the actual source may exhibit up to ± 20% non-uniformity in activity distribution, as allowed by ICRU recommendations [3]. Figure 1 shows the mathematical eye phantom along with ^106^Ru/^106^Rh eye plaque.

**Fig. 2:**
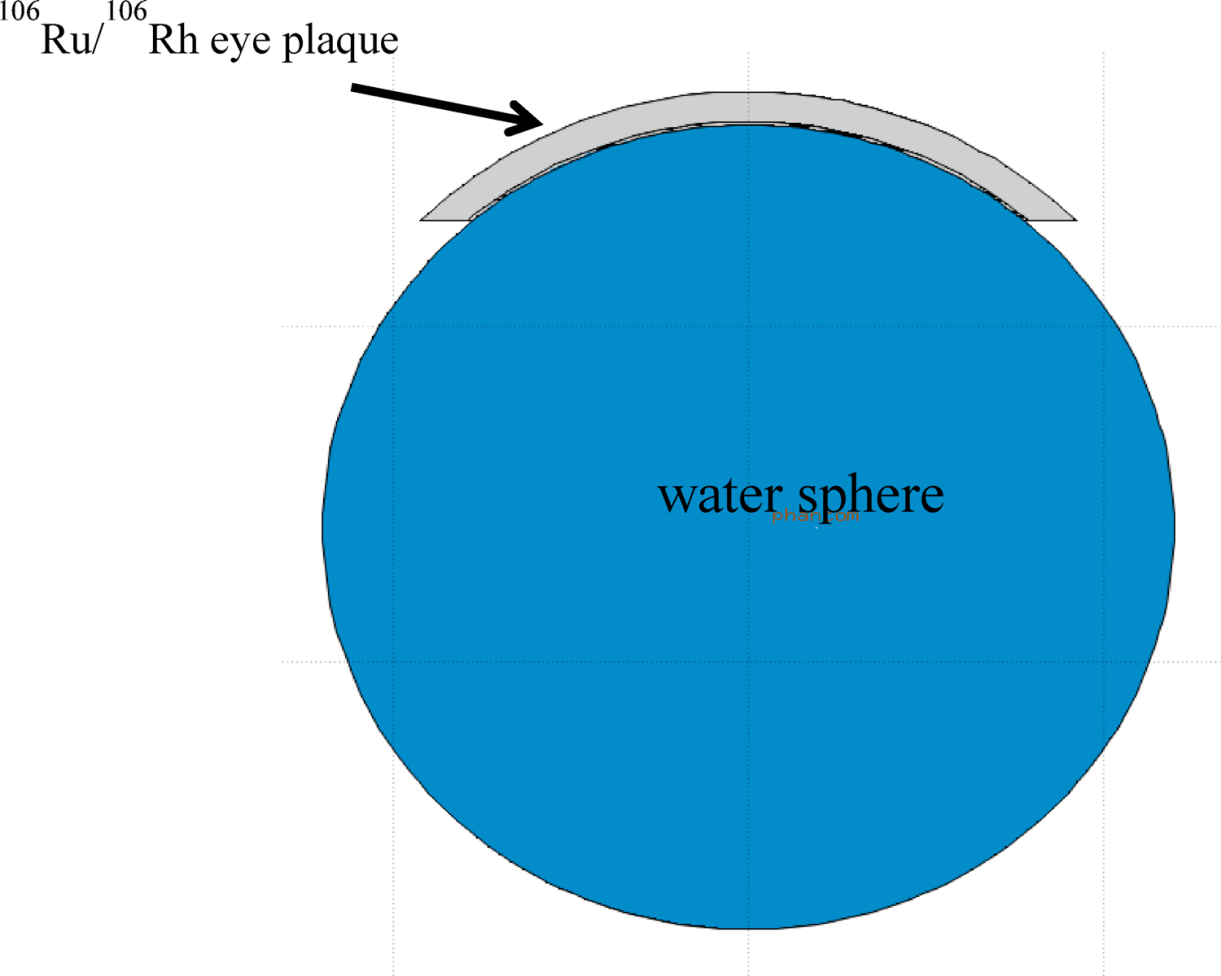
Water phantom with ^106^Ru/^106^Rh eye plaque in FLUKA

A spherical water phantom was also simulated by declaring all the materials of mathematical eye phantom as water. In order to compare the calculated dose results, a spherical water phantom (density: 1 g/cm^3^) along with ^106^Ru/^106^Rh eye plaque was also simulated (see Fig. 2). Note that the eye plaque is in contact geometry with mathematical and water phantoms in the simulations.

In the present study, all the simulations used EM-CASCA default card. The production and transport cut-off for both electrons and photons are set at 1 keV everywhere using EMFCUT card. Single scattering at the boundary is activated using MULSOP card. All the FLUKA-based Monte Carlo simulations described below were carried out in a 64-bit Ubuntu 20.04 LTS OS-based HP Z820 Work-station, which has an Intel^®^ Xeon (R) CPU E5-2650 @ 2.60 GHz processor and 32 GB RAM. The total number of processors is 32.

#### Dose calculations

On-axis absorbed doses to medium as a function of depth in mathematical eye and water phantoms *D*_*m*_(d) for CCA BEBIG and BARC eye plaques are calculated within1 mm x 1 mm x 0.25 mm voxels using USRBIN card. Note that medium “m” represents water in the case of water phantom and local medium in the case of mathematical phantom. *D*_*m*_(d) is expressed in mGy/particle. *D*_*m*_ is then converted dose-rate per MBq (mGy/min-MBq) using the following formula:

1 $$\:{\stackrel{\prime }{D}}_{m\left(d\right)}={D}_{m}\left(\mathrm{d}\right)\mathrm{x}\:\:k$$

Where *k* is a conversion coefficient and is given below:

2 $$\:k={10}^{6}\left(\frac{disintegrations}{s}\right)\times\:2\left(\frac{betaparticles}{disintegration}\right)\times\:60\left(\frac{s}{min}\right)$$

Using the depth doses, percentage depth dose(PDD) values are calculated by normalizing the dose values at 2 mm depth. In addition, off-axis dose profiles at different on-axis depths, 2D isodose distribution and organ doses are calculated for both the eye plaques in mathematical eye phantom. In the case of water phantom, percentage depth dose, on-axis and off-axis dose profiles are calculated. The scoring voxel sizes used for off-axis dose profiles and 2D isodose distribution are 1 mm x 0.25 mm x 0.2 mm and 0.25 mm x 0.25 mm x 0.2 mm, respectively. Organ doses to eye lens, retina, choroid and cornea, absorbed dose to medium are calculated using the USRBIN scoring card considering whole organ as detector region. The uncertainty associated with the dose estimate is only statistical. The 1σ statistical uncertainties on absorbed dose values are kept below 0.5%.

## 3. Results

### 3.1 On-axis depth dose profile

**Fig. 3:**
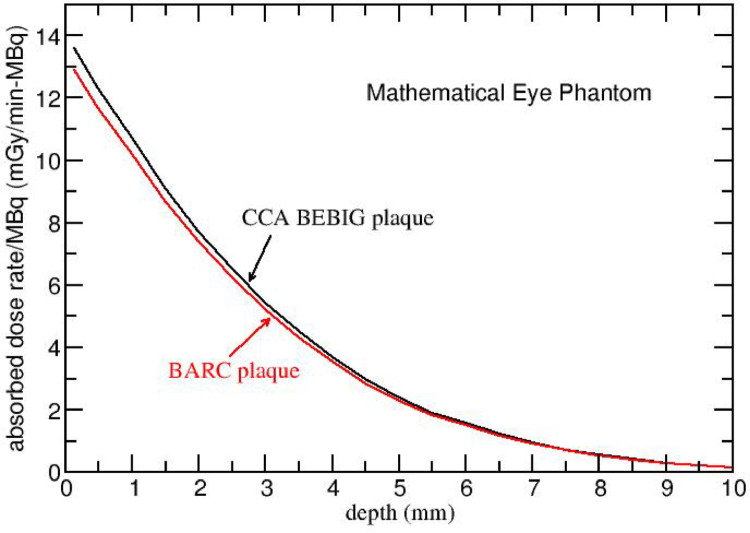
FLUKA-calculated absorbed dose rate per unit activity (mGy/min-MBq) for ^106^Ru/^106^Rh CCA and BARC eye plaques in mathematical eye phantom presented as a function of on-axis depth

Figure 3 presents the on-axis absorbed dose rate per MBq (mGy/min-MBq) as a function of depth (up to 10 mm) in the mathematical eye phantom for CCA and BARC eye plaques. The absorbed dose rate for CCA BEBIG plaque is systematically higher as compared to that for BARC plaque (within 4 – 7%). For example, the absorbed dose rate for CCA BEBIG plaque is 13.59, 10.71 and 7.72 mGy/min-MBq at 0.125 mm, 1 mm and 2 mm on-axis depths, respectively. Whereas it is 12.90, 10.19 and 7.39 mGy/min-MBq for BARC plaque at 0.125 mm, 1 mm and 2 mm, respectively.

**Fig. 4:**
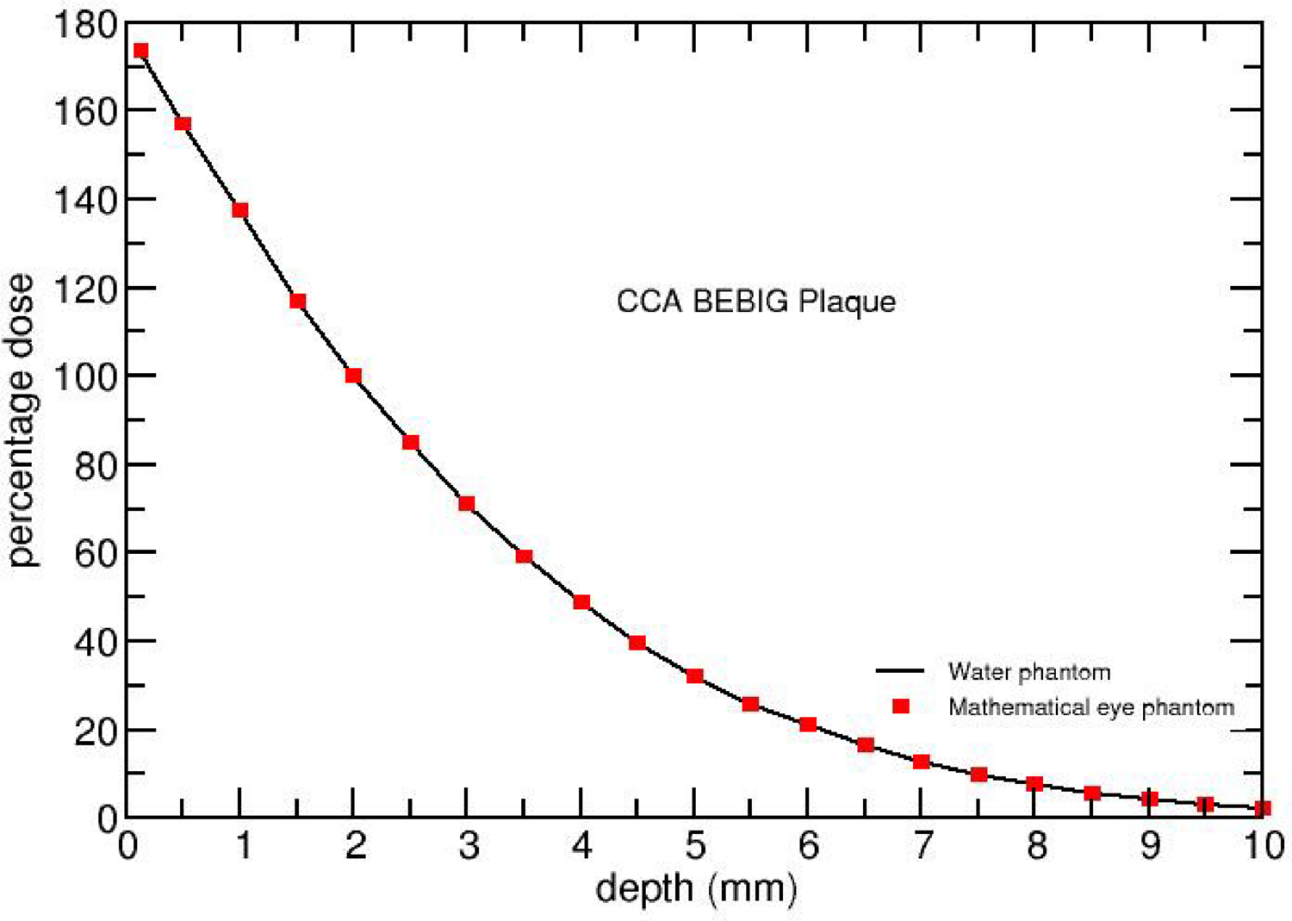
Comparison of FLUKA - calculated on-axis percentage dose for ^106^Ru/^106^Rh CCA eye plaques in water and mathematical eye phantom. The normalisation is done with respect to the dose value at 2 mm depth

**Fig. 5:**
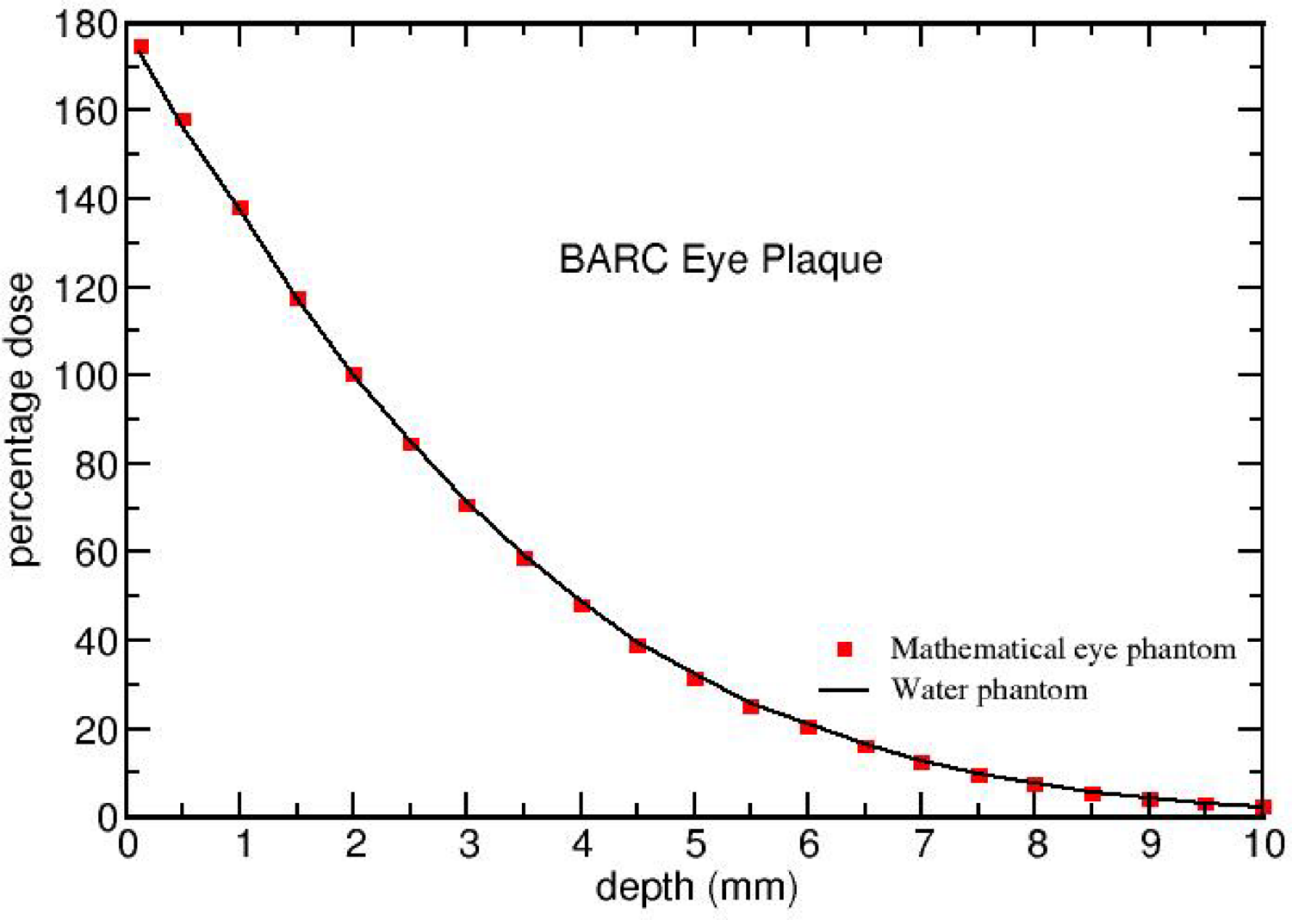
Comparison of FLUKA-calculated on-axis percentage dose for ^106^Ru/^106^Rh BARC eye plaques in water and mathematical eye phantom. The normalisation is done with respect to the dose value at 2 mm depth

Figures 4 and 5 compares FLUKA-calculated on-axis PDD of CCA and BARC ^106^Ru/^106^Rh eye plaques in water and mathematical eye phantoms. It shows that the PDD’s for both the eye plaques in both the materials (water and tissue) are in good agreement. This indicates that the water can also serves as a good approximation for dosimetry instead of various tissue composition.

### Absorbed dose rate

The calculated values of absorbed dose rate to water $\:\left({\stackrel{\prime }{D}}_{w}\right)$ at on-axis depths of 1 mm and 2 mm in the spherical water phantom for the CCA BEBIG eye plaque are 10.865 ± 0.005mGy/min-MBq and 7.906 ± 0.003 mGy/min-MBq, respectively. For the same eye plaque, the calculated values of absorbed dose rate to tissue ($\:{\stackrel{\prime }{D}}_{t}$) at on-axis depths of 1 mm and 2 mm in the mathematical eye phantom are 10.712 ± 0.003 mGy/min-MBq and 7.719 ± 0.002 mGy/min-MBq, respectively. For the BARC eye plaque, the Monte Carlo-calculated values of $\:{\stackrel{\prime }{D}}_{t}$ are10.194 ± 0.003 mGy/min-MBq and 7.392 ± 0.003 mGy/min-MBq at on-axis depths of 1 mm and 2 mm in the mathematical eye phantom, respectively.

The comparable values of $\:{\stackrel{\prime }{D}}_{w}$ and corresponding $\:{\stackrel{\prime }{D}}_{t}$ suggest that the dose rate is insensitive to both, i.e. tissue and water medium. FLUKA-calculated $\:{\stackrel{\prime }{D}}_{w}$ value at 2 mm depth for the CCA eye plaque at in water phantom is comparable (within 1.12%) with the corresponding Monte Carlo-based value of 8 mGy/min-MBq reported by Barbosa et al.[12].

### Off-axis dose profiles

**Fig. 6:**
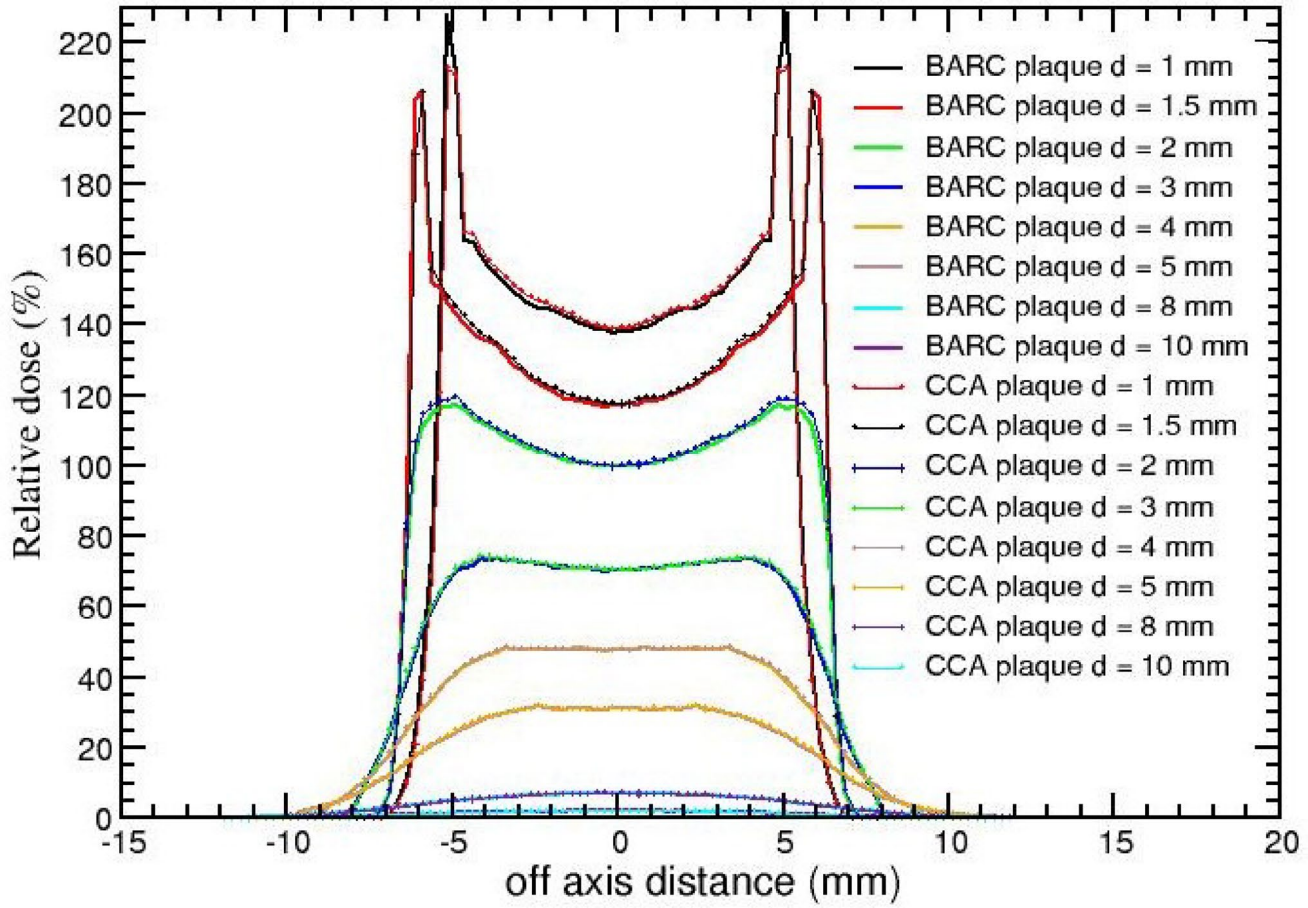
FLUKA-calculated relative dose (%) presented as a function of off-axis distance for BARC and CCA^106^Ru/^106^Rh eye plaque in the mathematical eye phantom. The normalisation is done with respect to the on-axis dose value at 2 mm depth

Figure 6 illustrates the calculated relative off-axis dose distributions at various on-axis depths (1, 1.5, 2, 3, 4, 5, 8, and 10 mm) in the mathematical eye phantom for the BARC and CCA eye plaques, respectively. These dose values have been normalized to 100% at the on-axis depth of 2 mm. The figure displays a symmetrical increase in relative dose values with off-axis distance, particularly at shallower depths (1 and 1.5 mm). At a depth of 1 mm, sharp peak is shown at the off-axis distance of 5.125 mm for both the plaques and the relative dose values are 228% and 212% for BARC and CCA eye plaques, respectively. Similarly, at the on-axis depth of 1.5 mm, the peak occurs at about 5.875 mm off-axis distance for both eye plaques.

### 2D dose mapping or isodose distribution

**Fig. 7:**
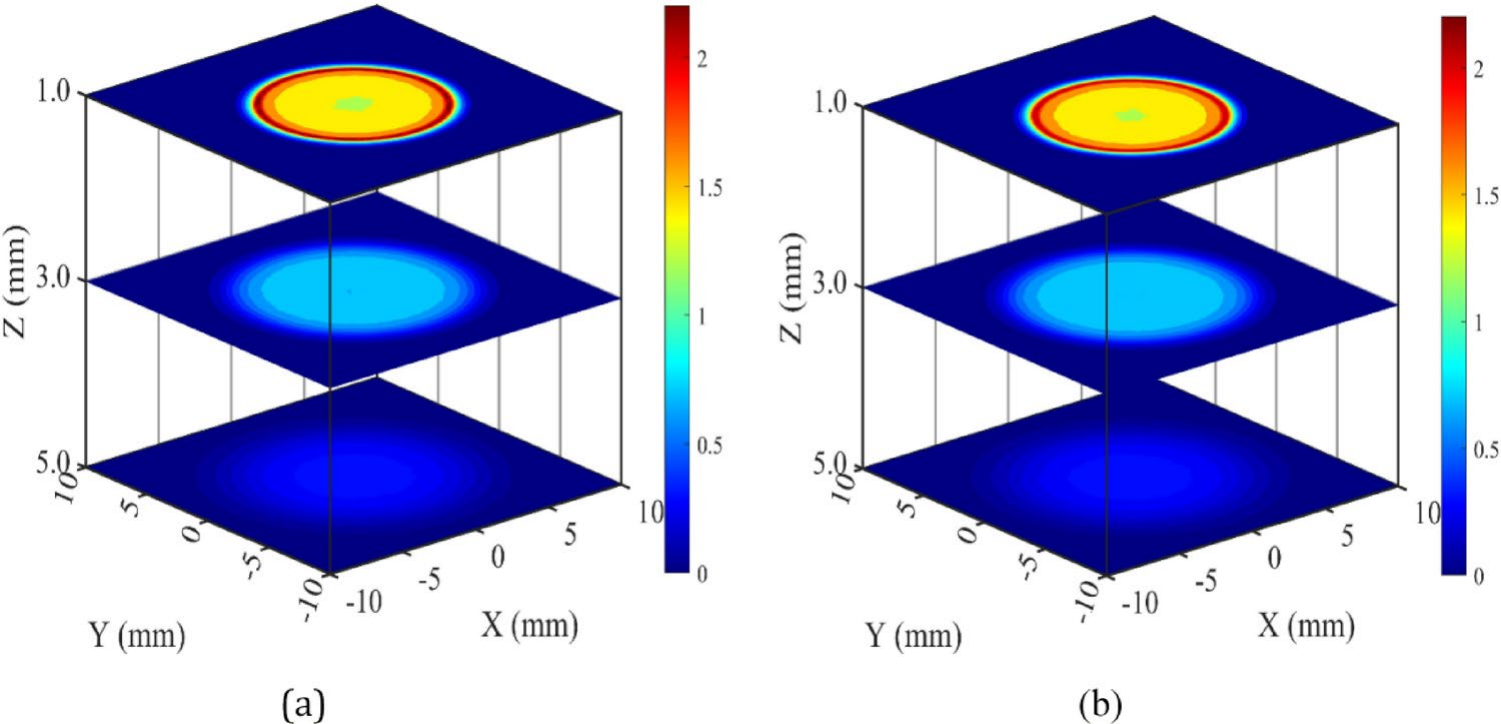
FLUKA-calculated isodose distributions of ^106^Ru/^106^Rh eye plaques in the mathematical eye phantom: **a** BARC eye plaque and **b** CCA eye plaque. Dose values are normalised with respect to the on-axis dose value at 2 mm depth

In Figs. 7(a) and (b), the 2D dose distributions are depicted at on-axis depths of 1 mm, 3 mm and 5 mm in the mathematical eye phantom for the BARC and CCA eye plaque. All the plots are presented along X and Y axes, covering a lateral distance ranging from − 10 mm to + 10 mm at 1 mm, 3 mm and 5 mm on-axis depths. Dose profiles are normalized with respect to on-axis dose value at 2 mm depth. These figures provide a comprehensive visualization of how the radiation dose is distributed at different depths and lateral distances. The areas covered by 95%, 90% and 50% isodose lines at 1 mm depth are 157.44 mm^2^, 157.63 mm^2^ and 158.44 mm^2^, respectively for BARC eye plaque. Whereas the same are 157.44 mm^2^, 157.5 mm^2^ and 158.38 mm^2^, respectively for CCA eye plaque. At 1 mm depth the lateral area covered by 95%, 90% and 50% isodose lines for CCA is almost similar to area covered by BARC eye plaque.

Figures 7(a) and (b) compares the planar dose distribution of BARC and CCA eye plaques at on-axis depth of 1 mm, 3 mm and 5 mm in mathematical eye phantom. These dose values are normalised with respect to the on-axis dose value at 2 mm depth of each plaque separately.

### Organ dose

For the given tumour position (see Fig. 1) in the simulated mathematical eye model for both the ^106^Ru/^106^Rh eye plaques, organ doses are calculated in the eye lens, retina, choroid, cornea and sclera using FLUKA. The calculated values of absorbed dose rate (mGy/hr-MBq) for both the eye plaques are presented in Table 2. On comparing the organ dose values of both the plaques, it is clear that the BARC eye plaque is reproducing the same dose distribution as of CCA BEBIG eye plaque. Depending on tumour size and location, the typical prescribed dose for uveal melanoma is between 80 and 100 Gy at the tumour apex [12]. To deliver 80 Gy at tumour apex (which is at 5 mm in present study) the doses received by eye lens, retina and cornea are also presented in Table 2 for both plaques. Table 2 also includes dose tolerance values for eye lens, retina, and cornea specified by RTOG (Radiation Therapy Oncology Group) document [26]. Thus, the doses received by the eye lens, retina, and cornea are well within the organ dose tolerance [26] during the delivery of 80 Gy dose at the tumour apex. Note that for choroid and sclera no tolerance doses are given by RTOG.

**Table 2:** Density and elemental composition of each eye region [12]

Sclera, Choroid, Retina, Cornea, and Tumour	Percent by weight	Lens	Percent by weight	Vitreous body, Anterior chamber and Water	Percent by weight
ρ = 1.04 g/cm^3^		ρ = 1.07 g/cm^3^		ρ = 1 g/cm^3^	
˂Z/A > = 0.551		˂Z/A > = 0.547		˂Z/A > = 0.555	
H	10.454	H	9.6	H	11.2
C	22.663	C	19.5	O	88.8
N	2.490	N	5.7	-	-
O	63.525	O	64.6	-	-
Na	0.112	Na	0.1	-	-
Mg	0.013	P	0.1	-	-
Si	0.030	S	0.3	-	-
P	0.134	Cl	0.1	-	-
S	0.204	-	-	-	-
Cl	0.133	-	-	-	-
K	0.208	-	-	-	-
Ca	0.024	-	-	-	-
Fe	0.005	-	-	-	-
Zn	0.003	-	-	-	-
Rb	0.001	-	-	-	-
Zr	0.001	-	-	-	-

## Discussion

### On-axis depth dose profile

Figures 4 and 5 present that the PDD’s are in good agreement for water and tissue for both the eye plaques. Note that the dose distribution in a material depends on electron density as well as on mass density of the medium through which the beta particles are traversing. The electron density of a material is proportional to Z/A value of that material, where Z represents the atomic number and A the atomic mass number of the material. The effective Z/A value, $\:{\left(Z/A\right)}_{eff}$ is 0.555 for water and 0.551 for other eye materials (Sclera, Choroid, Retina, Cornea, and Tumour), The $\:{\left(Z/A\right)}_{eff}$ of water is only 0.7% higher than that of other organs and the mass density of eye materials is nearly equal to that of water (see Table 3). Hence, the PDDs for both the eye plaques in water and the mathematical eye phantoms are comparable.

**Table 3:** Absorbed dose-rate (mGy/hr-MBq) and absorbed dose (Gy) in various organs

Organ	Absorbed Dose-Rate (mGy/hr-MBq)		Absorbed Dose (Gy)^a^		
	BARC plaque	CCA plaque	BARC plaque	CCA plaque	RTOG values [26]
Eye lens	6.599 ± 0.004	6.652 ± 0.003	3.821 ± 0.003	3.699 ± 0.002	4
Retina	26.155 ± 0.003	26.301 ± 0.003	15.147 ± 0.002	14.625 ± 0.002	45–50
Choroid	20.564 ± 0.001	20.736 ± 0.001	11.909 ± 0.001	11.531 ± 0.001	--
Cornea	0.717 ± 0.001	0.727 ± 0.001	0.415 ± 0.001	0.404 ± 0.001	40
Sclera	57.633 ± 0.049	61.267 ± 0.035	33.376 ± 0.028	35.481 ± 0.020	--

^a^ Cumulative absorbed dose received by organs for 80 Gy prescribed dose at tumour apex.

### Off-axis dose profiles

It is observed from Fig. 6 that as the off-axis distance increases, dose value for both the plaques increases. Since the points located off-axis at initial depths are at lesser distance from the inner surface of the plaque compared to the central axis point, they receive more dose. Due to the curvature of the plaque, the dose received by the tissue along the circumference is highest compared to the points located near the central axis. The off-axis profile (Fig. 6) for both plaques in mathematical eye phantom shows horns at the shallower depths such as 1 mm, 1.5 mm and 2 mm and thereafter it is smoothened. Hence at shallow depths the horns are more prominent. At 1 mm depth, the calculated dose value for BARC plaque is higher by 5 to 11% than CCA plaque at the off-axis distances of ± 5.125 mm to ± 6.625 mm on both sides. At 1.5 mm depth, for BARC plaque the calculated dose values are higher by 7 to 8% than CCA plaque at off-axis distances of 6.375 mm to 6.625 mm. Whereas at 2 mm depth the calculated dose values are more for CCA plaque than BARC plaque by 11 to 12% at ± 6.625 mm to ± 7.325 mm on both sides. At all depths these horns are observed in the silver region for which the atomic number and mass density are higher as compared to the eye itself. The same trend was observed by Sánchez-Reyes [2] and Hokkanen [27]. There may be two possible reasons due to the curved nature of the beta source for this trend; (a) the radiation intensity decreases inversely proportional to the distance squared and its effect is more pronounced for on-axis point than at any other point in the same plane, and (b) the exponential attenuation offered by the tissue will be more for on-axis point than the off-axis points at the same plane. Note that these horns do not have any clinical implication as they are observed in the plaque metal itself and do not appear in the eye tissue. However, beyond 1.5 mm, the dose distribution at the edges becomes smoother. However, the presence of off-axis horns may influence dosimetric verification depending on the detector used, particularly for devices with limited spatial resolution. The recommended detectors, such as radiochromic film and small plastic scintillators, possess sufficient spatial resolution to capture these steep dose gradients and minimize measurement artifacts.

### 2D dose mapping or isodose distribution

Figures 7(a) and (b) indicates that the dose distribution patterns of these two plaques are almost similar. But the dose values at various points specially near the edge of the plaque at same planes for both the plaques may differ slightly due to the slight variation in the plaque geometry.

### Organ dose

The doses received by the critical organs such as eye lens, retina, choroid, cornea and sclera are sensitive to the shape, size and position of the tumours as well as the position of the plaque. The mathematical modelling of an eye will provide additional information about the dose received by individual eye structure and also help in making the changes if any is required to obtain a suitable clinical treatment plan.

## Conclusions

The present study conducted FLUKA-Monte Carlo based comprehensive dosimetry for BARC ^106^Ru/^106^Rh eye plaque and a similar commercially available BEBIG CCA eye plaque in a heterogeneous mathematical eye phantom and homogenous water phantom. The calculated dosimetric parameters are absorbed dose rate, percentage depth dose, Off-axis dose profiles at different on-axis depths, and 2D dose mapping. The good agreement between the FLUKA-calculated reference absorbed dose rate for CCA plaque at 2 mm in water phantom and the corresponding published values demonstrates the accuracy and reliability of simulations. This study demonstrated that the BARC plaque delivers dose distributions comparable to the well-established BEBIG CCA plaque while maintaining safe dose levels for critical ocular structures. The agreement in key dosimetric parameters, including absorbed dose rates, PDD, and off-axis profiles, for BARC plaque with those for CCA plaque underscores the reliability and precision of the BARC plaque in clinical settings. The similarity in depth dose distributions between the mathematical eye phantom and the spherical water phantom suggests that water can serve as a reasonable approximation for dosimetry assessments. However, modelling a detailed mathematical eye phantom is essential for accurately evaluating the doses received by critical ocular structures. The 2D dose distributions calculated in mathematical eye phantom gives valuable information into dose distribution patterns at various depths and lateral distances.

## Data Availability

All data that supporting the findings of this study is included within the article.
